# Factors that influence the uptake of postnatal care from the perspective of fathers, partners and other family members: a qualitative evidence synthesis

**DOI:** 10.1136/bmjgh-2022-011086

**Published:** 2023-05-02

**Authors:** Kenneth Finlayson, Emma Sacks, Vanessa Brizuela, Nicola Crossland, Sarah Cordey, Daniela Ziegler, Etienne V Langlois, Dena Javadi, Liz Comrie-Thomson, Soo Downe, Mercedes Bonet

**Affiliations:** 1 School of Community Health and Midwifery, University of Central Lancashire, Preston, UK; 2 Department of International Health, Johns Hopkins School of Public Health, Baltimore, Maryland, USA; 3 Department of Reproductive Health and Research, World Health Organization, Geneva, Switzerland; 4 Direction de l'enseignement et l'Académie CHUM | Bibliothèque du CHUM, Centre Hospitalier de l'Universite de Montreal, Montreal, Québec, Canada; 5 Partnership for Maternal, Newborn and Child Health (PMNCH), World Health Organization, Geneva, Switzerland; 6 Department of Social and Behavioral Sciences, Harvard TH Chan School of Public Health, Boston, Massachusetts, USA; 7 Global Women's and Newborn's Health Group, Burnet Institute, Melbourne, Victoria, Australia

**Keywords:** Maternal health, Systematic review

## Abstract

**Background:**

Postnatal care (PNC) is a key component of maternity provision and presents opportunities for healthcare providers to optimise the health and well-being of women and newborns. However, PNC is often undervalued by parents, family members and healthcare providers. As part of a larger qualitative review exploring the factors that influence PNC uptake by relevant stakeholders, we examined a subset of studies highlighting the views of fathers, partners and family members of postpartum women.

**Methods:**

We undertook a qualitative evidence synthesis using a framework synthesis approach. We searched multiple databases and included studies with extractable qualitative data focusing on PNC utilisation. We identified and labelled a subset of articles reflecting the views of fathers, partners and other family members. Data abstraction and quality assessment were carried out using a bespoke data extraction form and established quality assessment tools. The framework was developed *a priori* based on previous research on the topic and adapted accordingly. Findings were assessed for confidence using the GRADE-CERQual approach and are presented by country income group.

**Results:**

Of 12 678 papers identified from the original search, 109 were tagged as ‘family members views’ and, of these, 30 were eligible for this review. Twenty-nine incorporated fathers’ views, 7 included the views of grandmothers or mothers-in-law, 4 incorporated other family member views and 1 included comothers. Four themes emerged: access and availability; adapting to fatherhood; sociocultural influences and experiences of care. These findings highlight the significant role played by fathers and family members on the uptake of PNC by women as well as the distinct concerns and needs of fathers during the early postnatal period.

**Conclusion:**

To optimise access to postnatal care, health providers should adopt a more inclusive approach incorporating flexible contact opportunities, the availability of more ‘family-friendly’ information and access to psychosocial support services for both parents.

**WHAT IS ALREADY KNOWN ON THIS TOPIC**

- Postnatal care can improve health outcomes for women and their babies but is sometimes overlooked or undervalued by women, their families and healthcare providers. Women’s access to postnatal care is often mediated or affected by influential family members.

**WHAT THIS STUDY ADDS**

- This is the first study to systematically explore and synthesise the views of key family members regarding their engagement with postnatal services. Our findings indicate that the informational, care and psychosocial support needs of influential family members, especially fathers, are not being fulfilled by healthcare providers, resulting in suboptimal engagement in some contexts. In addition to meeting these requirements, a more inclusive service incorporating flexible contact opportunities and respect for sociocultural norms may optimise postnatal care utilisation.

**HOW THIS STUDY MIGHT AFFECT RESEARCH, PRACTICE OR POLICY**

- To increase utilisation of postnatal care by women and their families carefully designed interventions need to adopt a more family-centred approach incorporating the needs of parents as well as influential family members where appropriate.

## Introduction

Postnatal care (PNC) is a key component of the maternity care continuum and contributes to improved health outcomes for women and infants.^1^ The postnatal period refers to the days and weeks following the birth of a baby and there is general consensus that this equates to the first 6 weeks after childbirth.^2^ Routine PNC is a formal service provision specifically designed to support, advise, inform, educate, identify those at risk and, where necessary, manage or refer women or newborns, to ensure optimal transition from childbirth to parenthood and childhood. Postnatal care can include a wide range of activities, including risk identification (assessments, screening), prevention of complications, health education and promotion and support for families.^3 4^

In its recently updated guidance, the WHO recommends that all women and babies receive PNC within 24 hours of childbirth, irrespective of where the birth occurs, followed by a minimum of three postnatal check-ups (either community or facility based) during the first 6 weeks.^3^ PNC within the first 24–48 hours is more likely to be facility based while subsequent care may be community based and, in some settings, may include home visits by healthcare providers.

The benefits of PNC are highlighted in The Global Strategy for Women’s, Children’s and Adolescents’ Health 2016–2030, which emphasises the importance of providing postnatal services.^5^ Estimates suggest that up to 27% of newborn deaths could be avoided if routine PNC and curative care reached 90% of mothers and their babies during the postnatal period.^6^ However, the postnatal period is often overlooked by women, families and health providers, with more resources and activities directed at the antenatal and intrapartum periods.^7^ Evidence from a recent analysis indicates that postnatal services have among the lowest median national coverage of interventions on the continuum of maternal and child healthcare.^8^ In addition, a recent analysis of PNC data from 48 low or lower middle income counties shows very poor PNC coverage following discharge from a health facility and large disparities between coverage of women and newborns.^9^ Utilisation of postnatal services in low and middle-income countries varies considerably both between and within countries with access affected by socioeconomic circumstances and living in an urban or rural location.^10^

Previous reviews exploring access to maternity services by women indicate that a variety of issues including socioeconomic, sociocultural and sociodemographic factors as well as previous encounters with maternity providers (either positive or negative) contribute to women’s engagement with antenatal, intrapartum and postnatal care.^11–13^ These reviews also highlight the influential role played by family members in the uptake of maternity services by women. In some contexts, where patriarchal societies may limit women’s ability to control family finances and healthcare access, the decision to engage with healthcare providers is often made by the male partner^14–16^ or by a senior female relative.^17^ Several studies have shown that engaging with influential family members, especially men, during the maternity phase can have a positive impact on women’s uptake of antenatal services and institutional delivery with a skilled birth attendant.^18–20^ In addition, interventions designed to engage men in maternal and newborn health have demonstrated an increase in care seeking, increased health-promoting home care practices and the development of more equitable couple communication and decision-making.^21^

As part of a larger qualitative review (henceforth termed the primary review), exploring the factors that influence the uptake of PNC by women and their families,^22^ we wanted to examine separately the views of other family members to understand their influence over women’s decision-making as well as their own views on engagement with postnatal services.

## Methods

We conducted a qualitative evidence synthesis utilising a framework approach and thematic synthesis techniques to analyse and synthesise relevant qualitative data.^23^ Descriptive themes were generated and organised into review findings that were assessed for confidence using the GRADE-CERQual tool.^24^

Studies investigating the views and opinions of family members of postnatal women were included. We defined family members as any person from the immediate family including fathers, partners, coparents, grandparents, siblings of the parents and aunts or uncles of the parents. We included studies reporting qualitative data from family members of women who gave birth in any setting (including a health facility or at home). Studies solely investigating experiences of, or beliefs about specific postnatal practices including breast feeding or infant feeding were excluded as were studied with a focus on specific conditions requiring specialist postnatal care. Inclusion and exclusion criteria are presented in table 1.

**Table 1:** Inclusion and exclusion criteria

Inclusion criteria for primary review	Exclusion criteria for primary review	Additional inclusion/exclusion criteria for the current analysis on family members’ views on routine postnatal care
Studies including healthy women, who were considered to be healthy in the postnatal period, and who have had a healthy newborn as well as fathers, partners and other family members Studies where at least some of the extractable data are women’s, and/or fathers/partners/and other family members own accounts of their views and experiences of the nature of, provision of, and/or seeking of postnatal care after birth, irrespective of parity, mode of birth or place of birth Studies involving postnatal care experiences with or without interaction with the health system (home-based, community-based care, care by family members) Studies from high, middle and low-income countries	Studies reporting on views/experiences of, or access to, maternity services generally with no specific data on postnatal care. Women with known complications/health conditions (eg, depression), or after severe morbidity (eg, near-miss) Services for specific conditions (eg, HIV), or high-risk populations (eg, multiples, preterm, low birth weight, malformations). Specific interventions for a singular condition (eg, breastfeeding support, family planning, mental health) or postnatal education only (eg, parenting education) Studies related to care of postnatal complications or intensive care for women or newborns Mixed-methods studies reporting qualitative data without using a recognised qualitative approach to analysis Case studies, conference abstracts and unpublished PhD or master’s theses Systematic reviews (although reference lists were reviewed)	*Inclusion* Studies including fathers/partners and other family members’ first-hand accounts (not reported through a third party) Studies focused on fathers/partners and other family members’ views of postnatal care *Exclusion* Studies focused on populations other than fathers, partners and other family members (eg, non-family community members, providers and traditional birth attendants (TBA) Studies reporting only on experiences of fatherhood rather than experiences of postnatal care

The search strategy and study selection methods of the primary review are summarised elsewhere.^22^ Briefly, the Qualitative Evidence Synthesis (QES) included qualitative or mixed methods studies that incorporated a qualitative component in the study design, data collection or method of analysis. Databases included MEDLINE (OVID), PubMed, CINAHL (EBSCO), EMBASE (OVID), EBM-Review (OVID) and a grey literature search via BASE (Bielefeld University Library), OpenGrey and the WHO website. The search strategy covered papers published from inception to December 2019. There were no language restrictions. Duplicates were removed through the EndNote X9 software using a method developed by Bramer *et al*.^25^

Records were collated into Covidence software and screened by title and abstract. Several members of the study team (ES, MB, VB) independently screened titles and abstracts against the inclusion/exclusion criteria and flagged studies that met both the general inclusion criteria and those that were specific to the views of family members. Any doubts regarding inclusion/exclusion were resolved by consensus among the authors.

### Data extraction and analysis

Data extraction, analysis and quality appraisal proceeded concurrently and broadly followed the ‘best fit’ framework approach described by Carroll *et al*^26^ and incorporating thematic synthesis techniques^23^ to develop new themes where emerging data failed to fit our *a priori* framework. Based on previous related reviews of antenatal care^27^ and intrapartum care,^28^ we used a deductive approach to develop a thematic framework which did not entirely match the topic under study but was a ‘best-fit’ and provided a relevant framework and themes against which to map and code the data from the studies identified for this review.^26^ The framework comprised four broad concepts (Resources and access; Behaviours and attitudes; External influences and What family members want and need) as well as a number of subthemes (see online supplemental appendix 1 for details of the initial framework). We began by using an Excel spreadsheet to record pertinent details from each study, for example, author, country, publication date, study design, setting, data collection methods, participant details, contexts, etc (see online supplemental appendix 2). The four concepts from our *a priori* framework were added to the Excel sheet and codes developed corresponding to the author-identified findings from each study and mapped to the framework as appropriate. Where author findings were presented as metaphors or abbreviated quotes, we developed codes based on our interpretation of the associated narrative. Coded material which did not align with the concepts from our *a priori* framework were labelled as ‘other’. At the end of the coding phase, these ‘other’ codes were examined by two reviewers (KF and SC) and new concepts and themes were developed via secondary thematic analysis, in accord with methods derived from the best fit approach.^26^

10.1136/bmjgh-2022-011086.supp1 Supplementary data

10.1136/bmjgh-2022-011086.supp2 Supplementary data

Most of the identified studies were published in English. Two studies were published in Portuguese, one in German, and one in Danish. Following further review by speakers of these languages at the WHO, three were excluded on the basis of relevance and only one Portuguese study was included. Data extraction and analysis of this paper were conducted by a team member (VB) proficient in Portuguese.

### Quality assessment

Included studies were appraised using an instrument developed by Walsh and Downe^29^ and modified by Downe *et al*.^30^ Studies were rated against 11 predefined criteria and then allocated a score from A–D (see online supplemental appendix 3 for details).

10.1136/bmjgh-2022-011086.supp3 Supplementary data

Studies were independently appraised by members of the review team and a 10% sample were cross-checked by a different study team member to ensure consistency. Any studies where there were scoring discrepancies of more than a grade were referred to a third team member for moderation. Studies scoring C or higher were included in the final analysis.

Following data extraction and quality appraisal, the framework codes, including any codes that emerged during the mapping phase, were grouped into descriptive themes.^23^ Once the framework of descriptive themes (or review findings) was agreed by all authors, the level of confidence in each review finding was assessed using the GRADE-CERQual tool^24^ and agreed by consensus between two study team members (KF and SC). GRADE-CERQual assesses the methodological limitations, relevance, coherence and adequacy of data supporting a review finding. Based on these criteria, review findings were graded for confidence using a classification system ranging from ‘high’ to ‘moderate’ to ‘low’ to ‘very low’. Following CERQual assessment, the review findings were grouped into higher order analytical themes and the final framework was agreed by consensus among the authors.

### Patient and public involvement

Patients and members of the public were not involved in the design and conduct of this review.

## Results

**Figure 1:**
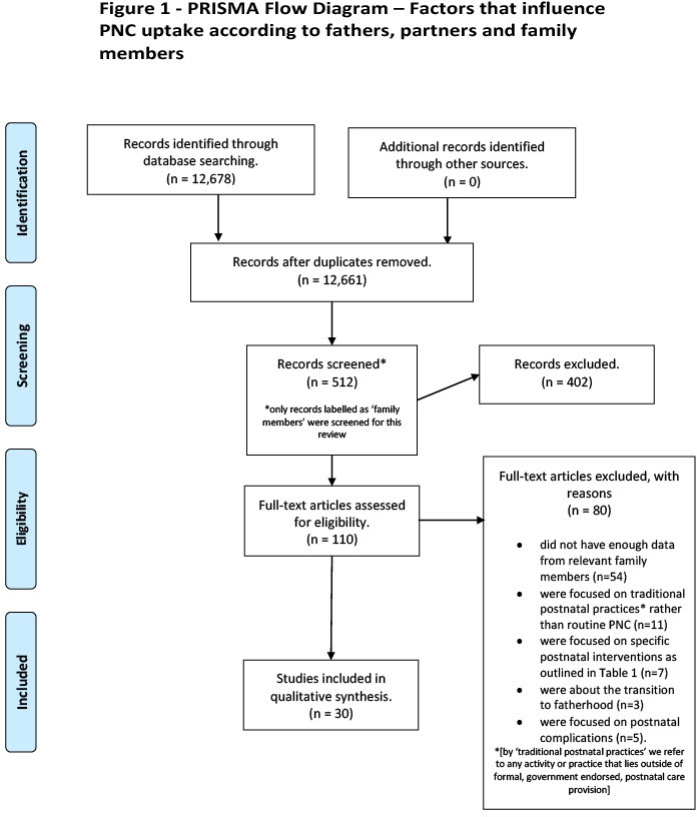
Preferred Reporting Items for Systematic reviews and Meta-Analyses (PRISMA) flowchart.

Our searches from the primary study yielded 12 678 records, from which 17 duplicates were removed. 12 149 records were excluded by title and by abstract, leaving 512 for full-text review. Of these, 110 were tagged as ‘family members’ and a further 80 were removed at this stage because they failed to meet the inclusion criteria (see figure 1—PRISMA flowchart for details).

Of the 30 included studies, 29 incorporated the views of fathers, 7 the views of grandmothers or mothers-in-law, 4 incorporated the views of other family members and 1 included comothers. Nine studies were focused on fathers only. The studies were conducted in a range of different settings including 12 from Low-income countries or lower middle-income countries (LMICs) and 19 from upper middle income countries or high-income countries (HICs), according to the 2021 World Bank classification. They represent a variety of global regions including Europe (n=11), Africa (n=8), Asia (n=5), North America (n=3), the Middle East (n=2) and South America (n=1). In total, there were more than 800 participants in the included studies, representing the views of more than 650 fathers, 170 mothers-in-law or grandmothers and 3 comothers. In terms of quality, 25 of the included studies were graded as A or B (+ or −) with five graded as C (+ or −). No studies were excluded on the basis of quality. Details of the study characteristics are shown in table 2.

**Table 2:** Characteristics of included studies

Author(s) and title	Year	Country (income level)	Context	Study design	Participants and location/focus of study	Quality rating
Al Tarawneh, *et al*.^59^ ‘*Being relieved and puzzled’: A qualitative study of first-time fathers’ experiences postpartum in Jordan*	2019	Jordan (upper middle)	Urban—employees at a public university	Qualitative and exploratory informed by semi-structured interviews	12 first-time fathers. All aspects of PNC	B
Amare, *et al*.^60^ *Early postnatal home visits: a qualitative study of barriers and facilitators to achieving high coverage*	2018	Ethiopia (low)	Rural—healthcare districts in South-East Ethiopia	Qualitative and exploratory using interviews and focus group discussions (FGD)	25 interviews and 4 FGDs with mothers, 4 FGDs with fathers and 4 with grandmothers. Home visits by community health workers	B
Barimani M & Vikstrom A.^61^ *Successful early postpartum support linked to management, informational, and relational continuity*.	2015	Sweden (high)	Urban—participants recruited from antenatal clinics in a Swedish city	Qualitative and exploratory using FGDs	7 FGDs with 18 mothers and 16 fathers. Facility based care and home visits	B
Danbjorg DB, *et al*.^62^ *Do families after early postnatal discharge need new ways to communicate with the hospital? A feasibility study*.	2014	Denmark (high)	Urban—postnatal ward of a city hospital	Participatory design process derived from Action Research and framed with critical theory	7 interviews with parents plus 1 FGD with primiparous parents (n=5), and 1 FGD with multiparous parents (n=4) including 5 fathers. Facility-based care and home visits	B
de Montigny F & Lacharite C.^63^ *Fathers’ Perceptions of the Immediate Postpartal Period*.	2003	Canada (high)	Urban—postnatal ward of a city hospital	Qualitative and descriptive using semi- structured interviews	13 first-time fathers. Facility-based care	B+
de Oliveira EMF & de Brito RS.^64^ *Actions in care carried out by the father in the puerperism (Ações de cuidado desempenhadas pelo pai no puerpério*).	2009	Brazil (upper middle)	Urban—in a large city hospital in Brazil (Natal)	Qualitative and descriptive using semi structured interviews	15 fathers. Facility-based care	C+
Fredriksson GEM, *et al*.^65^ *Postpartum care should provide alternatives to meet parents’ need for safety, active participation, and ‘bonding*.	2003	Sweden (high)	Urban—on a hospital midwifery ward and a family suite	Qualitative and descriptive (part of a larger qualitative study exploring different kinds of maternity care), using semi structured interviews	11 couples and one mother, including both first-time and experienced parents. Facility-based care in a ‘family suite’ and at home following discharge	B
Gaboury J, *et al*.^66^ *Effect of the Postpartum Hospital Environment on the Attainment of Mothers’ and Fathers’ Goals*.	2017	Canada (high)	Urban hospital—participants recruited from postnatal ward	Qualitative and descriptive informed by interviews	10 women (of various parities) and 8 partners interviewed separately in the immediate post-partum phase (within 48 hours). Facility-based care	A-
Grant M, *et al*.^67^ *Trust of community health workers influences the acceptance of community-based maternal and child health services*	2017	South Africa (upper middle)	Rural—8 clinics in 5 rural districts in KwaZulu-Natal province	Qualitative and exploratory informed by FGDs	19 FGDs in total including 3 with 21 fathers and 3 with 19 grandmothers of children<5. Home-based care by community health workers	B
Gupta ML, *et al*.^58^ *Grandmothers as gatekeepers? The role of grandmothers in influencing health-seeking for mothers and newborns in rural northern Ghana,*	2015	Ghana (lower middle)	Rural—a rural district in the North East region of Ghana	Qualitative and exploratory using interviews and FGDs (conducted with a wide variety of stakeholders)	35 interviews with mothers and 18 FGDs including 8 with grandmothers (n=81). Community clinics and home-based care	B
Henshaw EJ, *et al*.^68^ ‘*Trying to Figure Out If You’re Doing Things Right, and Where to Get the Info’: Parents Recall Information and Support Needed During the First 6 weeks Postpartum*.	2018	USA (high)	Urban—an urban centre in Ohio using flyers and online methods	Qualitative and exploratory based on grounded theory, informed by FGDs with parents	5 FGDs with 26 mothers, 5 fathers and 1 sister of young children. All aspects of PNC	A-
Hunter L.^69^‘*The views of women and their partners on the support provided by community midwives during postnatal home visits.’*	2004	UK (high)	Urban and rural—sample recruited by community midwives working for a National Health Service Trust serving urban and rural populations	Qualitative and exploratory utilising a grounded, hermeneutical approach and informed by interviews	5 cohabiting couples (5 fathers) up to 6 weeks after birth. All first-time parents. Home-based care by midwives	B-
Johansson M, *et al*.^70^ *Fathers want to stay close to their partner and new baby in the early postnatal period: the importance of being able to room in after a surgical birth*.	2013	Sweden (high)	Urban—a regional city hospital in Northern Sweden	Qualitative and descriptive, informed by telephone interviews with fathers whose partners had experienced a caesarean section	21 fathers of babies born by elective or emergency caesarean section. Facility-based care	C+
Kurth E, *et al*.^71^ *Safe start at home: what parents of newborns need after early discharge from hospital—a focus group study*.	2016	Switzerland (high)	Urban and sub-urban—in the region of Basel	Qualitative and descriptive using a ‘playful’ design informed by focus groups	24 participants in 6 FGDs including 5 with mothers (n=20) and 1 with partners (n=4) conducted up to 9 months postpartum. Home-based care	A-
Mbekenga CK, *et al*.^72^ *Postpartum experiences of first-time fathers in a Tanzanian suburb: a qualitative interview study*.	2011	Tanzania (lower middle)	Urban—recruited via a health clinic in a suburb of Dar es Salaam	Qualitative and descriptive informed by interviews	10 first-time fathers from a variety of different ethnic groups. All aspects of PNC	B+
Memon Z, *et al*.^73^ *Residual Barriers for Utilization of Maternal and Child Health Services: Community Perceptions From Rural Pakistan*.	2015	Pakistan (lower middle)	Rural—10 ‘socio-economically underdeveloped’ districts in Sihdh province	Qualitative and exploratory utilising an extensive network of FGDs with relevant stakeholders in 10 rural (hard to reach) areas of Pakistan	60 FGDs in total including 20 with mothers and 20 with fathers (approximately 10 participants in each group). All aspects of maternity care including PNC	B-
Newbrander W, *et al*.^74^ *Barriers to appropriate care for mothers and infants during the perinatal period in rural Afghanistan: a qualitative assessment*.	2013	Afghanistan (low)	Rural—small communities (close to and far from health facilities) in five disparate rural areas of Afghanistan	Qualitative and descriptive utilising interviews, FGDs and community observations	30 in-depth household interviews, 29 FGDs and 15 direct observations with women, their husbands, mothers-in-law, grandmothers and other family members. All aspects of maternity care including PNC	B-
Persson EK & Dykes AK.^75^ *Parents experience of early discharge from hospital after birth in Sweden*	2002	Sweden (high)	Urban—following discharge from maternity/family ward at the Helsingborg Hospital in southern Sweden	Qualitative and inductive using grounded theory for analysis	12 cohabiting parents (mix of parities), including 6 fathers whose babies were discharged home early (after 26 hours). Home-based care following early discharge	B+
Persson EK, *et al*.^76^ *Fathers' sense of security during the first postnatal week-a qualitative interview study in Sweden*.	2012	Sweden (high)	Urban—3 hospital uptake areas and 5 different postnatal wards in Southern Sweden.	Qualitative and inductive using interviews and FGDs, analysed using grounded theory	8 fathers in 3 FGDs and 5 interviews (mix of first time and 2nd/3rd time fathers). Facility and home-based care during first week	B
Probandari A, *et al*.^77^ *Barriers to utilization of postnatal care at village level in Klaten district, central Java Province, Indonesia*.	2017	Indonesia (lower middle)	Rural—Central Java province	Qualitative and exploratory informed by interviews	8 mothers, 6 family members (no details) and 5 midwives. Focus on barriers to all aspects of PNC engagement	B
Raven JH, *et al*.^78^ *Traditional beliefs and practices in the postpartum period in Fujian Province, China: a qualitative study*.	2007	China (upper middle)	Urban and rural—one rural and one rapidly developing urban county in Fujian Province.	Qualitative and descriptive utilising interviews	12 mothers, 12 husbands and 12 grandmothers of babies born in last 4 months. Focus on beliefs associated with PNC	B-
Ross NJ, *et al*.^79^ *The Perspectives of Young Men and Their Teenage Partners on Maternity and Health Services During Pregnancy and Early Parenthood*.	2012	UK (high)	Urban—public health context around teen parenting	Qualitative, exploratory and longitudinal (data collected by interview in late pregnancy and 18 months postpartum)	30 young couples including men aged 16–24 and women aged 16–19. All aspects of maternity care including PNC	C
Reuben Mahiti G, *et al*.^80^ *Perceptions about the cultural practices of male partners during postpartum care in rural Tanzania: a qualitative study*.	2017	Tanzania (lower middle)	Rural—Kongwa district of the Dodoma region, located in central Tanzania.	Qualitative and descriptive, informed by FGDs	14 FGDs with a total of 93 men. Focus on beliefs associated with PNC	B
Sharkey A, *et al*.^81^ *Maternal and newborn care practices in Sierra Leone: a mixed methods study of four underserved districts*.	2017	Sierra Leone (low)	Rural—4 relatively remote areas of Sierra Leone (Kambia, Tonkolili, Kailahun and Pujehun).	Mixed methods—household cluster survey plus in-depth interviews and FGDs	Grandmothers or aunties (16 interviews; 8 FGDs) and fathers (23 interviews; 8 FGDs). Focus on beliefs associated with PNC engagement	A-
Shorey S, *et al*.^82^ *Lived experiences of Asian fathers during the early postpartum period: Insights from qualitative inquiry*.	2018	Singapore (high)	Urban—a tertiary public hospital in Singapore.	Qualitative and descriptive informed by interviews	50 first-time and experienced fathers. Facility-based care	A-
Simbar M, *et al*.^83^ *Fathers' educational needs for perinatal care in urban Iran: a qualitative approach*.	2010	Iran (lower middle)	Urban—4 city hospitals	Qualitative and descriptive informed by FGDs	8 FGDs (n=46 participants) including 4 FGDs with fathers (n=22). Focus on engagement with PNC	C+
Solberg B & Galvin K.^84^ *Fathers want to play a more active role in pregnancy and maternity care and at the child health centre*.	2018	Norway (high)	Urban—a medium-sized municipality in South East Norway	Qualitative and descriptive informed by semi-structured interviews	13 interviews with first-time fathers. All aspects of maternity care including PNC	B
Tesfaye G, *et al*.^85^ *Delaying factors for maternal health service utilization in eastern Ethiopia: A qualitative exploratory study*.	2020	Ethiopia (low)	Urban and Rural— Kersa district, Eastern Ethiopia.	Qualitative and interpretive using FGDs	3 FGDs with mothers-in-law (n=19) and 3 with fathers (n=24). Focus on barriers to all aspects of PNC engagement	A
Vikström A & Barimani M.^86^ *Partners' perspective on care-system support before, during and after childbirth in relation to parenting roles*.	2016	Sweden (high)	Urban—a large Swedish city	Qualitative and exploratory informed by FGDs	3 FGDs with fathers (n=14) and 1 with co-mothers (n=3) including 12 first-time parents. All aspects of maternity care including PNC	C+
Zamawe CF, *et al*.^87^ *The role of the parents’ perception of the postpartum period and knowledge of maternal mortality in uptake of postnatal care: a qualitative exploration in Malawi*.	2015	Malawi (low)	Unclear. An area in central Malawi with high levels of poverty and low uptake of postnatal care services	Qualitative and descriptive informed by interviews and focus groups	1 FGD with 14 partners (fathers) plus FGDs with 36 first-time mothers up to 1 year postpartum. Focus on beliefs associated with PNC engagement	B

PNC, postnatal care.

### Findings

We generated 17 descriptive themes (review findings), including five that were assessed as ‘high’ confidence, seven as ‘moderate’ and five as ‘low’ confidence. These descriptive themes were mapped against our *a priori* framework themes to generate our final analytical themes. *Resources and Access* was retained; *Behaviours and Attitudes* was changed to ‘*Adapting to Fatherhood’* to better reflect the attitudes and beliefs of male partners in the included studies. We altered *External influence*s to *Socio-cultural Influences* to incorporate the particular societal concerns that impacted on behaviours, and we changed *What Family Members Want and Need* to *Experience of Care* to better reflect the experiential nature of the findings.

A summary of the four analytical themes as well as the relevant descriptive themes, supporting quotes and CERQual assessments are shown in table 3.

**Table 3:** Review findings and CERQual assessments

Theme	Subtheme	Supporting quote	Contributing papers	CERQual grade
**Resources and access**	**Importance of home visits:** Fathers from studies largely conducted in HICs highlighted the importance of regular home visits by health providers, both for themselves and for their partner. Some fathers felt that these visits were particularly important following early discharge home and a few felt that more frequent and/or longer home visits (> once per week) would be beneficial. Fathers also felt that home visits should be flexible, unhurried, and responsive to parental needs rather than standardised, provider-led appointments.	‘Then I thought it was good to have a home visit from the well children’s clinic. They came home and checked things. It felt very good. I could almost wish that there were more of those check-ups’. (Father), Persson, 2012, (Sweden)	6 Studies: Al Taranweh, 2019 (Jordan); Fredriksson, 2003 (Sweden); Hunter, 2004 (UK); Kurth, 2016 (Switzerland); Persson, 2002 (Sweden); Persson, 2012 (Sweden)	Moderate
	**Flexible contact opportunities**: Fathers and co-mothers in studies from several HICs indicated that more flexible contact arrangements with healthcare providers had/would have a positive impact on their own engagement with PNC services. First-time fathers in particular felt that a 24-hour service or a dedicated hotline would be beneficial and emphasised the importance of being able to speak to a health provider at night.	‘In the beginning it is good if one has somebody 24-hours a day, something like a telephone hotline, a central office.’ (Father), Kurth, 2016 (Switzerland). ‘My understanding is that you should try to have this kind of daily contact even if there isn’t anything special that’s happening. I don’t think we had any special questions, just the feeling of security if we did have a question’. (Father), Fredriksson, 2003, (Sweden).	5 Studies: Barimani, 2015 (Sweden); Fredriksson, 2003 (Sweden); Hunter, 2004 (UK); Kurth, 2016 (Switzerland); Persson, 2002 (Sweden)	Moderate
	**Facility environment**: Fathers and family members in several settings highlighted facility based organisational and environmental issues that impacted on their engagement with postnatal care. These included a lack of staff at the health facility which occasionally raised safety concerns as well as long waiting times at visits. A few family members also discussed the importance of the facility environment in terms of cleanliness and/or privacy while others highlighted the disruptive nature of hospital routines (ward rounds, visiting hours, alarms going off) as a factor in their desire to leave the ward early. In one setting the perceived poor quality of publicly funded health facilities prompted parents to seek expensive private care.	‘It’s not a very nurturing look. If the environment was more welcoming then it soothes you. This is our house for these 3 days’. (Father), Gaboury, 2017, (Canada). ‘Women in the village inform each other about the waiting time to receive the service after birth and discourage themselves from receiving the service’ (Mother-in-law), Tesfaye, 2019, (Ethiopia)	7 Studies: de Montigny, 2004 (Canada); Fredriksson, 2003 (Sweden); Gaboury, 2017 (Canada); Memon, 2015 (Pakistan); Persson, 2012 (Sweden); Solberg, 2018 (Norway); Tesfaye, 2019 (Ethiopia).	Low
	**Poor transport networks**: Fathers and mothers-in-law from studies in isolated LMIC settings highlighted distance from a health facility and concerns with the road networks, topography and associated transport difficulties as potential barriers to PNC access for women and for themselves.	‘Since Bajaj [tricycle vehicle] and ambulances can’t enter the village because of the difficult road for vehicles, it would be good if the roads connecting the village to the main road are constructed…’ (Mother-in-law), Tesfaye, 2019, (Ethiopia)	5 Studies: Amare, 2018 (Ethiopia); Memon, 2015 (Pakistan); Newbrander, 2013 (Afghanistan); Tesfaye, 2019 (Ethiopia); Zamawe, 2015 (Malawi)	Low
**Adapting to fatherhood**	**Importance of fathers’ psychosocial needs**: Fathers in a variety of different settings reported difficulties in coping with the postnatal period. Fathers experienced feelings of exhaustion, loneliness, insecurity, anxiety and inadequacy and sometimes felt ill-equipped to offer psychological and emotional support to their partners. In many cases they found it difficult to access support to meet their own psychosocial needs and some felt that provider-organised peer support groups would be beneficial.	‘I have no idea what to do in the postpartum…never had such an experience before…I felt I need support too’. (Father), Al Tarawneh, 2019 (Jordan). ‘It’s challenging and difficult to broach topics related to this [fathers mental health] … what you feel.’ (Father), Solberg, 2018 (Norway).	11 Studies: Al Tarawneh, 2019 (Jordan); de Montigny, 2004 (Canada); Fredriksson, 2003 (Sweden); Hunter, 2004 (UK); Johansson, 2013 (Sweden); Kurth, 2016 (Switzerland); Mbekenga, 2011 (Tanzania); Ross, 2012 (UK); Simbar, 2010 (Iran); Solberg, 2018 (Norway); Vikstrom, 2016 (Sweden)	High
	**Concerns about the financial impact of a new baby**: Fathers and family members from a variety of different settings expressed concerns about the financial burden associated with having a new baby. For some fathers the arrival of a baby placed even more pressure on limited family finances and restricted contact with PNC services because of the costs associated with attendance (transport and medicine). For other fathers the financial responsibility of providing for an extra family member prompted them to work harder or take on more work which had a detrimental effect on their own ability to engage with PNC services.	‘The husband is expected to provide for the family since there is expenditure every day. So, a husband must continue to produce to cater for household expenditure.’ (Mother-in-law), Rueben Mahiti, 2017 (Tanzania). ‘Money is definitely a thing that made me feel insecure, that we haven’t enough money. It’s just that feeling of taking care of the family in some way’. (Father), Persson, 2012 (Sweden).	8 Studies: de Oliveira, 2009 (Brazil); Gupta, 2015 (Ghana); Mbkenga, 2011 (Tanzania); Memon, 2015 (Pakistan); Newbrander, 2013 (Afghanistan); Rueben-Mahiti, 2017 (Tanzania); Persson, 2012 (Sweden); Zamawe, 2017 (Malawi)	High
	**Willingness to embrace new responsibilities**: Fathers, from studies predominantly conducted in high and middle-income settings, often expressed a willingness to become involved in supporting their partner and new baby during the postnatal period. Sometimes this was expressed as a sense of responsibility or duty and a recognition that they may need to advocate for their partner/wife during contact with postnatal services, even if this meant breaking with social norms. For some fathers the limited amount of paternity leave and/or the demands of work-related commitments curtailed their involvement in post-natal activities (including care-seeking) and caused frustration.	‘So, she (partner) needs more of my attention. Just five days is too short a time to provide help to her. I think that (…) ten days (…) would be ideal.’ (‘Então ela precisa de mais atenção da gente. Só cinco dias é pouco tempo pra dar assistência a ela. Acho que (…) dez dias (…) seria ideal.’—(Father) de Oliveira, 2009 (Brazil).	9 Studies: Al Tarawneh, 2019 (Jordan); de Oliveira 2009 (Brazil); Fredriksson, 2003 (Sweden); Johansson, 2013 (Sweden); Kurth, 2016 (Switzerland); Persson, 2002 (Sweden); Shorey, 2018 (Singapore); Simbar, 2010 (Iran); Vikstrom, 2016 (Sweden)	Moderate
**Socio-cultural influences**	**Influence of decision maker**: In a number of studies from LMICs, the decision to engage with formal postnatal services was often taken by a family member (husband, mother-in-law, or grandmother) or occasionally a community elder. In circumstances where formal postnatal care was appreciated and valued by the decision maker, engagement was authorised or encouraged, but in several instances, women were prohibited or discouraged from visiting facility based postnatal services by influential family members.	‘If the baby is sick and the father is not around, you must send him [the baby] to hospital. So we take such decisions when it comes to baby’s health.’ (Grandmother), Gupta, 2015 (Tanzania).	8 Studies: Gupta, 2015 (Tanzania); Mbkenga, 2011 (Tanzania); Newbrander, 2013 (Afghanistan); Raven, 2007 (China); Rueben-Mahiti, 2017 (Tanzania); Simbar, 2010 (Iran); Tesfaye, 2019 (Ethiopia); Zamawe, 2015 (Malawi)	High
	**Influence of socio-cultural norms and practices**: In studies conducted in a variety of different LMICs fathers and family members highlighted a range of established norms and practices that impacted on women’s engagement with formal PNC services. PNC practices about 'doing the month' or limiting interactions outside of the household during the postnatal period meant that women were unable to visit a health facility or receive attention from health workers for a prescribed amount of time. Local postnatal practices, the preference to see a Traditional birth attendant (TBA), and potentially harmful superstitious beliefs about the cause of postnatal problems, also limited women’s interactions with formal health providers. In other settings fathers, highlighted a tension between adhering to established postnatal practices and a desire to engage with formal approaches to post-natal care.	‘We are not certain if the foreign way can be done here. It is not that we don't believe in it, we just don't want to try that way. So if we can follow the traditional way, we just follow it.’ (Husband), Raven, 2007 (China).	10 Studies: Al Taranweh, 2019 (Jordan); Grant 2017 (South Africa); Mbkenga, 2011 (Tanzania); Newbrander, 2013 (Afghanistan); Probandari, 2017 (Indonesia) Raven, 2007 (China); Rueben-Mahiti, 2017 (Tanzania); Sharkey, 2017 (Sierra Leone); Simbar, 2010 (Iran); Tesfaye, 2019 (Ethiopia).	High
	**Lack of awareness about the benefits of PNC**: In studies conducted in several LMIC settings fathers and family members’ sometimes held a view that engagement with formal postnatal care was unlikely to be beneficial and/or that interaction with formal PNC services should only occur in the event of an emergency. In some of these settings there was also a tendency to prioritise antenatal care over postnatal care or prioritise the needs of the baby over the concerns of the woman which limited women’s access in these contexts.	‘…After giving birth it is not important for the woman to go a health facility or no need for check-up unless she faces health problems, because she is healthy, and the newborn is also fine…’ (Husband), Tesfaye, 2019 (Ethiopia).	5 Studies: Memon, 2015 (Pakistan); Probandari, 2017 (Indonesia); Rueben-Mahiti, 2017 (Tanzania); Tesfaye, 2019 (Ethiopia); Zamawe, 2015 (Malawi)	Moderate
	**Perceptions of masculinity**: In some studies from LMIC settings societal beliefs relating to masculinity affected men's willingness to become involved in post-natal care. In these contexts men believed that post-natal care was women's business and their involvement would be viewed as servile, controlling or shameful by the local community. The perception of postnatal care as women’s business was also evident in some husbands’ reluctance for their wife to be seen by a male care provider.	‘I want to share with my wife the care of our new baby, but in reality it is difficult. Imagine if [people] see me helping my wife with bathing the baby! They would tell me that such acts are shameful and that I should avoid them.’ (Father), Al Tarawneh, 2019 (Jordan).	6 Studies: Al Tarawneh 2019 (Jordan); Grant, 2017 (South Africa); Mbkenga, 2011 (Tanzania); Rueben-Mahiti, 2017 (Tanzania); Simbar, 2010 (Iran); Tesfaye, 2019 (Ethiopia);	Low
**Experiences of care**	**Need for information**: In a wide variety of settings and contexts, fathers (particularly first-time fathers) highlighted their need for information and advice about postnatal care. Usually, this related to practical skills around infant care needs (recognising crying cues, feeding, bathing, signs of distress) and many fathers highlighted the importance of having this information delivered to both parents at the same time. Some fathers felt unprepared prior to leaving a hospital after delivery and thought that information about the postnatal period should be given during the antenatal phase. Other fathers felt that there should be more information tailored to fathers’ needs and/or there should be more use of technology to convey information (e.g. phone apps, dedicated web pages).	‘I would have liked more advice on how to care for a newborn baby. To be told what to do at every stage of development. We are not informed…’. (Father), Mbkenga, 2011 (Tanzania). ‘If something is wrong, they should tell us without our having to push for the information’ (Father), de Montigny, 2004 (Canada).	18 Studies: Al Tarawneh, 2019 (Jordan); Barimani, 2015 (Sweden); Danbjorg, 2014 (Denmark); de Montigny, 2004 (Canada); de Oliveira, 2009 (Brazil); Fredriksson, 2003 (Sweden); Gaboury, 2017 (Canada); Henshaw, 2018 (USA); Johansson, 2013 (Sweden); Kurth, 2016 (Switzerland); Mbkenga, 2011 (Tanzania); Persson, 2012 (Sweden); Ross, 2012 (UK); Sharkey, 2017 (Sierra Leone); Shorey, 2018 (Singapore); Solberg, 2018 (Norway); Vikstrom, 2016 (Sweden); Zamawe, 2015 (Malawi)	High
	**Being included/excluded**: Some fathers felt included in conversations with health providers and, in one context, were given priority in a queueing system if they visited a health facility with their partner. However, a larger majority felt ignored or excluded in their interactions with providers. Sometimes this was experienced non-verbally, i.e. attention was directed at the mother only, whilst in other settings fathers were made to feel unwelcome and even prohibited from attending PNC appointments with their partner.	‘I wasn’t excluded by nurses, they didn’t ask me to leave the room, but it was a nonverbal exclusion, by the way their body was…they never asked me how I felt as a dad.’ (Partner) de Montigny, 2004 (Canada). ‘Perinatal services are provided in a female environment and we cannot attend’. (Unlabelled), Simbar, 2010 (Iran).	11 Studies: de Montigny, 2004 (Canada); Gaboury, 2017 (Canada); Hunter, 2004 (UK); Johansson, 2013 (Sweden); Mbkenga, 2011 (Tanzania); Persson, 2002 (Sweden); Ross, 2012 (UK); Reuben-Mahiti, 2017 (Tanzania); Simbar, 2010 (Iran); Solberg, 2018 (Norway); Vikstrom 2016 (Sweden)	Moderate
	**Need for reassurance**: Fathers in studies from some HICs expressed a desire to have their contributions to postnatal care recognised by health providers. They wanted to be asked questions by midwives or health visitors and to have their concerns addressed with understanding and reassurance.	‘It was important to me that all the involvement I had had during pregnancy, childbirth and now, after, be recognized by someone else than my spouse’. (Father), de Montigny, 2004 (Canada). ‘I mean the high point for me was just them saying … ‘yes, you're both doing fine’. (Partner), Hunter, 2004 (UK)	9 Studies: Barimani, 2015 (Sweden); Danbjorg, 2014 (Denmark); de Montigny, 2004 (Canada); Hunter, 2004 (UK); Kurth, 2016 (Switzerland); Persson, 2002 (Sweden); Persson, 2012 (Sweden); Shorey, 2018 (Singapore); Vikstrom, 2016 (Sweden)	Moderate
	**Importance of provider attitude and behaviour**: Fathers and family members in a variety of different settings described how the attitude of healthcare providers could influence their engagement with postnatal services. Staff displaying personal qualities of care and compassion and the ability to empower parents were viewed favourably whilst healthcare providers who were perceived to be disrespectful, judgemental, untrustworthy or professionally incompetent were viewed negatively and a potential deterrent to parental engagement with postnatal services.	‘Staff attitude is also not good with patients and that is why people do not prefer to visit the RHC [Reproductive Health Centre].’ (Father), Memon, 2015 (Pakistan). ‘It doesn’t matter if they are highly skilled if they don’t have the personal qualities to build relationships.’ (Father), Solberg, 2018 (Sweden)	10 Studies: Amare 2018 (Ethiopia); Danbjorg, 2014 (Denmark); Gaboury, 2017 (Canada); Grant, 2017 (South Africa); Hunter, 2013 (UK); Memon, 2015 (Pakistan); Persson, 2002 (Sweden); Probandari, 2017 (Indonesia); Ross, 2012 (UK); Solberg, 2018 (Norway);	Moderate
	**Continuity of care**: Fathers in studies from several HICs indicated that it was important to build a relationship with a known midwife, ideally one that had been present at the birth of their baby. Some expressed frustration at having to repeatedly recite information to different health professionals during the post-natal phase.	‘It would be good if one already knew in pregnancy who the midwife will be, who’s coming for postpartum visits.’ (Partner), Kurth, 2016 (Switzerland).	5 Studies: Barimani, 2015 (Sweden); Hunter, 2004 (UK); Kurth, 2016 (Switzerland); Persson, 2002 (Sweden); Vikstrom, 2016 (Sweden)	Low
	**Inconsistent advice**: Fathers in studies from some HICs expressed frustration at the inconsistent and occasionally conflicting advice given to them about postnatal care practices by health providers. This was particularly apparent in discussions around the support they could give to their wife/partner experiencing difficulties with breastfeeding.	‘It’s like, even though you’re somewhere where everyone’s supposed to be an expert on breastfeeding they all say different things about what to do when things get difficult’ (Father), Persson, 2012 (Sweden)	6 Studies: Barimani, 2015 (Sweden); Henshaw, 2018 (USA); Hunter, 2004 (UK); Kurth, 2016 (Switzerland); Persson, 2012 (Sweden); Vikstrom, 2016 (Sweden)	Low

### Resources and access

Fathers and other family members in several LMIC settings highlighted poor road networks and lack of public transport as potential barriers to PNC access. This was particularly apparent in rural locations where engagement with PNC could be limited by inaccessible topography, poorly maintained roads and unaffordable transport. Family members indicated that, in these locations, mothers were often unable to reach health facilities or health professionals were unable to reach communities.

In a number of settings, family members were disappointed by the poor staffing levels at health facilities and the subsequent lengthy waiting times during PNC visits. Fathers from different income settings were also disappointed by the poor state of repair at some health facilities and the lack of privacy in busy or overcrowded rooms. Fathers also commented on the disruptive nature of postnatal hospital wards and how visiting hours, ward rounds, persistent monitoring and alarms impacted on their ability to provide comfort and support to their partner and new baby.

Fathers and other family members discussed the importance of flexible contact opportunities during the postnatal period. Fathers in HICs often felt they needed support and reassurance at various times of the day or night and some wanted a 24-hour service that gave them the opportunity to contact a health professional (by phone, text or e-mail) when the need arose.

In addition, fathers also felt that more home visits would have a positive impact on their engagement with PNC. Some fathers indicated that they would prefer weekly home visits and/or open-ended encounters with an emphasis on individual needs rather than standardised provider-led procedures.

### Adapting to fatherhood

Fathers generally expressed a willingness to embrace their perceived responsibilities. They expressed a desire to bond with their baby, to become involved in infant-related feeding and bathing activities and, when required, to act as an advocate for their partner during the immediate postnatal period. For some fathers, this willingness to fulfil their paternal responsibilities was occasionally stifled by a perceived inadequacy in their paternity leave allowance or work commitments, which sometimes led to feelings of frustration or resentment.

In a variety of different contexts, fathers discussed the financial implications of having a baby and the impact this had on their ability to engage with postnatal services. Some fathers felt a pressure to provide (financially) for a new baby, which prompted them to take on more work. This often had a detrimental effect on their capacity to spend time with their family, to bond with their infant, and to attend postnatal visits/appointments, irrespective of their desire to do so. For other fathers, particularly in LMICs, the arrival of a new baby placed additional pressure on already stretched family finances and, with limited employment options, fathers sometimes felt they could not afford the extra costs associated with visiting a health facility for PNC.

The accumulation of different expectations and parental responsibilities sometimes had a negative impact on fathers’ emotional and psychological well-being. First-time fathers in particular reported feeling anxious and insecure in their new role and sometimes placed their partners’ support needs above their own, to the detriment of their own health. Occasionally, fathers indicated that they felt unable to support their partners or ill-equipped to do so which, in turn, led to feelings of inadequacy or anxiety.

In many instances, fathers felt that there was a lack of postnatal support services to address their social and psychological needs while also acknowledging that their partners’ well-being needs should be prioritised. To address these issues, fathers in some HIC settings suggested that providers could organise and/or facilitate peer support groups exclusively for fathers who might be struggling during the postnatal period.

### Sociocultural influences

In a number of LMIC contexts, women’s ability to engage with postnatal services was influenced and, in many cases determined, by other members of the family. In these contexts, societal beliefs relating to healthcare decision-making fell to husbands or grandmothers whose own views about the relative importance or need for PNC affected women’s engagement. However, societal beliefs relating to masculinity sometimes had a negative impact on fathers’ willingness to engage with postnatal activities since maternity care was viewed as an exclusively female environment where male engagement was considered to be servile or even shameful. Some studies demonstrated that these views extended to fathers’ beliefs relating to caregivers and a perception that women should not be seen by a male health professional. Nevertheless, in some LMIC contexts, there was a perception that the traditional gender roles associated with maternity care were beginning to wane, particularly among younger generations of men. Fathers in these settings sometimes highlighted a conflict between traditional concepts of masculinity and modern approaches to parenting, including their engagement with PNC.

In a variety of different LMIC settings, family members placed limited value on the benefits of formal PNC and/or were motivated by a desire to uphold traditional postnatal practices which resulted in poor engagement by women. The wish to maintain established approaches to PNC was sometimes reflected in the practice of ‘doing the month’ or similar activities that limited a new mother’s ability to engage with the outside world. In other settings, family members highlighted a range of beliefs that prompted them to seek help from a TBA or traditional healer rather than a formal healthcare provider. Furthermore, the value placed on formal PNC by family members had a direct impact on uptake and, in some LMIC settings, there was a perception that formal postnatal services should only be used in an emergency, for example, to treat a postnatal haemorrhage or a neonatal infection or fever. There was also a tendency to prioritise the other components of maternity care (antenatal and intrapartum), at the expense of PNC which was viewed as incidental or the least important element of maternity care.

### Experiences of care

Fathers in a wide variety of settings expressed a desire for more information and advice about PNC. This largely related to practical guidance on infant-related activities like feeding and bathing as well as support in recognising the signs of infant distress or crying cues. In some contexts, fathers felt that this kind of information should be delivered to both parents at the same time to aid mutual understanding, while in others, fathers felt that information should be tailored to their own needs by emphasising what fathers could do to support the well-being of mother and baby. Fathers also reported feeling unprepared after leaving a health facility and suggested it would be useful to receive information about the postnatal period prior to the birth, ideally during the antenatal phase.

In some HIC settings, fathers also expressed concerns about the inconsistency of advice given by health professionals, particularly in relation to their support of breastfeeding wives or partners. Fathers in these contexts also highlighted their frustrations with having to repeatedly recite personal details and queries to different health professionals. In some instances, they bemoaned the lack of continuity or stressed the importance of being able to build a trusting relationship with a known health professional. This was particularly important for some fathers in HICs who needed reassurance that their baby was developing normally or recognition that they were doing things correctly.

Both fathers and grandmothers highlighted specific provider qualities as an important element in their own willingness to engage with postnatal services as well as their perception of women’s willingness to do so. Where providers were perceived to be caring, compassionate and empowering, family members felt more at ease but when health professionals were viewed as disrespectful, judgemental or, in some cases, incompetent, they were seen as untrustworthy and a potential deterrent to further engagement.

Similarly, for fathers in all income level settings, provider characteristics which were perceived as exclusionary discouraged their engagement with postnatal services. In some LMIC contexts, this was overt and imposed, whereby fathers were actively prohibited from attending postnatal appointments, while in other contexts, the sense of exclusion was more subtle and reported as a mother-focused bias or a feeling of being ignored. Conversely, some fathers also described feeling included and acknowledged when health professionals directed attention to them and, in most instances, responded positively to the invitation to become more involved.

## Discussion

According to fathers and other family members, the factors that influence uptake of PNC are wide ranging and multifaceted. Our four key findings highlighting resources and access, adaptation to fatherhood, sociocultural beliefs and experiences of care, reflect a variety of practical, personal, societal and health system considerations that impact on PNC engagement by fathers and family members.

Our finding relating to resources and access highlights a persistent issue in maternity care engagement^12 28^ and, in this instance, a key consideration for fathers and family members in low-income settings where inadequate or expensive transport options limited PNC engagement. While previous reviews exploring barriers to maternity care access highlight poor infrastructure and transportation costs, the focus invariably falls onto antenatal and intrapartum care with little attention paid to PNC engagement.^31^ Similarly, demand-side initiatives including cash initiatives/subsidies and community engagement tend to prioritise antenatal and intrapartum care at the expense of PNC.^32^ While the rationale for these approaches is quite rightly framed around the desire to reduce maternal and neonatal mortality and morbidity, the emphasis on these aspects of maternity care may undermine the relevance and significance of PNC for fathers, family members and women.

Our finding around the influence of sociocultural practices contains insights that support the notion of PNC as an underused and/or overlooked aspect of maternity care, particularly in LMICs. It is, therefore, important that resources and interventions aimed at increasing women’s uptake of ANC or facility-based childbirth by targeting influential family members also focus on PNC utilisation. While interventions like the Birth Preparedness and Complication Readiness programme^33^ may lead to more facility-based births and subsequent facility-based PNC^34 35^ new interventions focusing specifically on increasing PNC utilisation are needed, especially in settings and communities where influential family members act as gatekeepers. Interventions may be particularly useful in settings where family members advocate an adherence to potentially harmful postnatal care practices or do not recognise the benefits of formal postnatal services. However, efforts to increase PNC utilisation through community-based education programmes aimed at fathers or couples in LMICs have had limited success.^21 36^ Some authors argue that ‘gender-transformative’ approaches, that is, using interventions that ‘actively examine and promote the transformation of harmful gender norms and seek to reduce inequalities between men and women to achieve desired outcomes’^37^ are more likely to be effective.^38 39^ Our finding related to ‘perceptions of masculinity’ suggests that, in some settings, younger generations of fathers may be open to these insights. Studies incorporating gender transformative approaches have been largely limited to reproductive healthcare, domestic violence and HIV transmission contexts^39^ although the gender transformative approach forms a key component of ‘The Fathers Club Manual’ a MenCare sponsored multicountry initiative aimed at engaging men in maternal, newborn and child health.^40^

From a supply-side perspective, our findings highlight the importance of home visits, particularly in HIC settings, as an opportunity for fathers and family members to become more involved in PNC and to have their own queries and concerns addressed. These findings resonate with evidence from other studies, which suggest that fathers feel more comfortable discussing their concerns with health professionals in their own homes and are better able to develop a father–infant bond under these circumstances.^41 42^ The recently published WHO recommendations on maternal and newborn care for a positive postnatal experience support this view and recommend home visits during the postnatal period across all care settings.^3^

Our key finding relating to ‘adaptation to fatherhood’ contains some unique insights into PNC utilisation from the perspective of fathers. In a variety of different settings, fathers expressed a willingness to become involved in PNC activities but often cited a lack of support in this regard. At a societal level, there are ongoing legal, ethical and cultural discussions about the nature, duration and benefits of paternity leave, but it is apparent that restrictive policies inevitably curtail PNC engagement by fathers. Social policies offering fathers extended periods of parental leave during the postnatal phase have proved successful in some Scandinavian contexts.^43 44^ However, in other settings, where extended/shared leave schemes are available, evidence suggests that prevailing attitudes towards gender roles, financial concerns and workplace culture can limit fathers’ engagement with PNC services (and mother and infant support) even when legal allowances are in place.^45 46^

With regards to our finding relating to experiences of care, we highlight a variety of paternal responsibilities and associated concerns (psychological, emotional and financial) that often go unacknowledged by healthcare providers. Fathers may feel obliged to adopt a supportive role, supressing or sacrificing their own emotional and psychological needs in favour of women’s and baby’s’ well-being. In this regard, our findings support a small but growing body of evidence highlighting an unmet requirement for more psychosocial support for fathers during the postnatal period.^47 48^ This may include targeted mental health support for fathers or partners who may be struggling with specific postnatal conditions like depression, post-traumatic stress disorder or anxiety^49^ or referral to fathers’ support groups or networks for fathers who may need additional social support or peer-to-peer connection.^50^

Along similar lines, our finding around inclusion/exclusion resonates with other studies in this area^51 52^ and suggests that more effort is required to actively engage with men during the postpartum period. This may be challenging in certain contexts particularly where heavy workloads and overcrowding impact on provider capacity to involve men or where lack of training on men’s needs and concerns or negative stereotypes of fathers inhibit provider engagement.^41^ Health professionals should be aware that although there appears to be a certain amount of ambivalence about how fathers perceive their role in terms of acting as support for mother and baby and being seen as a parent and individual with their own expectations and needs, acknowledgement of the latter is likely to lead to greater levels of satisfaction and an increased desire to engage with PNC services. Approaches using new technologies like mobile phone apps and online training may satisfy education and access needs in certain contexts, especially in remote communities where access to services may be challenging. Initial findings from several studies exploring digital health interventions and online forums with parents (or fathers only) during the postnatal period indicate that these platforms offer flexible contact opportunities and may be useful in facilitating PNC engagement by women and their families.^53–56^

These key findings closely resemble the themes identified by women in the primary review^22^ where access and availability; physical and human resources; external influences; social norms and experience of care were important influences on PNC uptake. Similarly, specific findings from this review relating to the need for information, reassurance and continuity as well as a desire to be cared for by respectful, empathic and compassionate staff are reflected in previous qualitative syntheses exploring women’s expectations and experiences of antenatal,^12 27^ intrapartum^28^ and postnatal care.^57^

### Strengths and limitations

While our review is framed around fathers and other family members, 29 of our 30 studies incorporated the views of fathers with more limited information coming from other family members, so our findings are heavily weighted towards fathers’ perceptions. Many of our included studies explored the views of fathers and other family members together, but only one study^58^ explicitly examined the views of other family members (grandmothers) and the scope of this study was across the maternity continuum rather than during the postnatal period specifically. This may limit our findings as the unique views of other family members may provide additional insights in contexts where influential family elders determine access to maternity care. Similarly, although our review identified two studies where same sex coparents (mothers) were included in the data collection phase, the views of the non-birthing partner were subsumed into the author-generated themes representing the wider body of participants (largely fathers), with little or no consideration of their differing circumstances or views. Additional research with these populations is likely to further enhance understanding of their own engagement with PNC and their influence on access by women.

In addition, some of our findings highlight issues and concerns that appear to be relevant to fathers, partners and other family members in certain contexts, for example, our subthemes relating to ‘flexible contact opportunities’, ‘the importance of home visits’, ‘need for reassurance’ and ‘continuity of care’ feature in HICs only, while ‘poor transport networks’, ‘influence of decision maker’, ‘perceptions of masculinity’, ‘lack of awareness about the benefits of PNC’ and ‘the influence of socio-cultural norms and practices’, relate primarily to LMICs. While we acknowledge that there may be context-specific issues, we are bound by the content of the included articles and recognise that different questions may have been posed to participants in different contexts, depending on the nature of the research inquiry and the pre-existing beliefs of the research team members.

One of the strengths of our review is the incorporation of a wide range of studies from different contexts around the world, including a mix of studies from high, middle and low-income settings. The data from the studies are relatively current, with all of the included articles being published within the last 20 years and over half published since 2015. We identified a very limited number of studies from Oceania and South America and information from fathers and family members in these regions is likely to add to the richness of the data and potentially contribute further insights beyond the findings from this review.

Our review offers some original insights into the perceptions of fathers and other family members regarding access to PNC services. They highlight a number of factors that could enhance engagement. Central to these findings is the need for a more inclusive service, which places the woman and baby at the heart of care but recognises and supports the significant role played by the wider family, particularly the father, during the postnatal period. Across a wide range of settings and contexts, PNC engagement is likely to be enhanced by a high-quality service offering inclusive, flexible contact opportunities with kind, respectful healthcare providers who appreciate and support infant well-being as well as the practical, informational and psychosocial needs of the entire family.

## Conclusion

Fathers and other family members identified a number of factors that influence uptake of PNC services by women as well as a variety of factors that affect their own engagement. In general, PNC engagement may be limited by a lack of resources, inability of PNC services to take account of sociocultural influences and limited information to fathers and family members about the potential benefits of formal PNC provided in a way that meets their needs and circumstances. The engagement of fathers may be further enhanced by adopting a more inclusive approach incorporating family-centred models of care as well as flexible contact opportunities, the availability of more father-focused information and access to psychosocial support services. There is also a need for more interventions aimed at engaging fathers and family members with a view to supporting PNC uptake, increasing effective coverage of PNC and promoting the integration and continuity of care across the maternity continuum. As with all components of maternity care, a recognition that high-quality care also embodies provider characteristics like competency, respect and kindness is likely to lead to more and sustained engagement with PNC.

## Data Availability

Data are available upon reasonable request.
