# Supplementary material for: Factors that influence the uptake of postnatal care from the perspective of fathers, partners and other family members: a qualitative evidence synthesis
Source: BMJ Glob Health. 2023 May 2;8(Suppl 2):e011086. doi: 10.1136/bmjgh-2022-011086 (PMC10163465; doi:10.1136/bmjgh-2022-011086)
Supplement: Supplementary data [file bmjgh-2022-011086supp001.pdf]

Appendix 2 - *A priori* framework

THEMES	POTENTIAL EXAMPLES
**RESOURCES AND ACCESS**	• Resource Availability - Could be a facilitator as well as a lack/barrier? i.e maternal resources, to access care, or resources at a facility or available to a visiting professional care giver<br>• Capacity to access health facility or healthcare provider? It could be quite close, but hard to get to, or a long way away, but easy to get to. In some systems, women don't go to health facility to access care, the healthcare provider comes to them<br>• Place of postnatal care/delivery arrangements (including issues around integration of mother and baby care, and issues around the question of home visits)<br>• Time/waiting times
**BEHAVIORS AND ATTITUDES**	• (Lack of) Respectful care<br>• (Lack of) trust in the system<br>• Belief (or not) in the need for postnatal care?<br>• Fear of stigma/test results/ policy/child services taking child away/knowing something is wrong with herself or the baby<br>• Value or otherwise of the mother/baby to the family/society (including not seeking care because babies are "weak", not considered "important", money not well spent if used for newborn care, etc.)
**EXTERNAL INFLUENCES**	• Influence of family/peers<br>• Capacity for women to travel for care/Freedom of movement/quarantine<br>• Influence of traditional/societal beliefs/superstitions (including social beliefs about postnatal care in modern and postmodern society that are not well grounded in evidence)
**WHAT WOMEN WANT AND NEED**	• Continuity of care/carer<br>• Need for information/advice (recognition of danger signs)<br>• Optimising health of the baby (including thriving and feeding)<br>• Optimising health of the mother (physical, psychological, emotional support)

	• Support for effective transition to motherhood (confidence, competence and adapting to changes in self and relationships to others)
