# Supplementary material for: Factors that influence the uptake of postnatal care from the perspective of fathers, partners and other family members: a qualitative evidence synthesis
Source: BMJ Glob Health. 2023 May 2;8(Suppl 2):e011086. doi: 10.1136/bmjgh-2022-011086 (PMC10163465; doi:10.1136/bmjgh-2022-011086)
Supplement: Supplementary data [file bmjgh-2022-011086supp003.pdf]

Appendix 3 – Quality Appraisal Tool

**Quality Score**	**Assessment Criteria**
A	Represents a study with no, or few flaws, with high credibility, transferability, dependability and confirmability
B	Represents a study with some flaws, unlikely to affect the credibility, transferability, dependability and/or confirmability of the study.
C	Represents a study with some flaws that may affect the credibility, transferability, dependability and/or confirmability of the study.
D	Represents a study with significant flaws that are very likely to affect the credibility, transferability, dependability and/or confirmability of the study.

* Some studies were graded as + or – (e.g. B+ or A-) depending on their conformity to the above criteria
